# Does vaporized hydrogen peroxide sterilization affect the geometrical properties of anatomic models and guides 3D printed from computed tomography images?

**DOI:** 10.1186/s41205-021-00120-w

**Published:** 2021-09-14

**Authors:** Mauricio Toro, Aura Cardona, Daniel Restrepo, Laura Buitrago

**Affiliations:** 1 TECHFIT Digital Surgery, Industrias Médicas Sampedro, Carrera 47 N° 100 Sur 40 Centro Industrial Portal del Sur, Bodega 14, variante a Caldas, La Estrella (Medellin), Colombia; 2 R&D Department, TECHFIT Digital Surgery, Industrias Médicas Sampedro, La Estrella, Colombia

**Keywords:** Computer-aided design, Printing, three-dimensional, Models, anatomic, Sterilization, Dimensional measurement accuracy

## Abstract

**Background:**

Material extrusion is used to 3D print anatomic models and guides. Sterilization is required if a 3D printed part touches the patient during an intervention. Vaporized Hydrogen Peroxide (VHP) is one method of sterilization. There are four factors to consider when sterilizing an anatomic model or guide: sterility, biocompatibility, mechanical properties, and geometric fidelity. This project focuses on geometric fidelity for material extrusion of one polymer acrylonitrile butadiene styrene (ABS) using VHP.

**Methods:**

De-identified computed tomography (CT) image data from 16 patients was segmented using Mimics Innovation Suite (Materialise NV, Leuven, Belgium). Eight patients had maxillary and mandibular defects depicted with the anatomic models, and eight had mandibular defects for the anatomic guides. Anatomic models and guides designed from the surfaces of CT scan reconstruction and segementation were 3D printed in medical-grade acrylonitrile butadiene styrene (ABS) material extrusion. The 16 parts underwent low-temperature sterilization with VHP. The dimensional error was estimated after sterilization by comparing scanned images of the 3D printed parts.

**Results:**

The average of the estimated mean differences between the printed pieces before and after sterilization were − 0,011 ± 0,252 mm (95%CI − 0,011; − 0,010) for the models and 0,003 ± 0,057 mm (95%CI 0,002; 0,003) for the guides. Regarding the dimensional error of the sterilized parts compared to the original design, the estimated mean differences were − 0,082 ± 0,626 mm (95%CI − 0,083; − 0,081) for the models and 0,126 ± 0,205 mm (95%CI 0,126, 0,127) for the guides.

**Conclusion:**

This project tested and verified dimensional stability, one of the four prerequisites for introducing vaporized hydrogen peroxide into 3D printing of anatomic models and guides; the 3D printed parts maintained dimensional stability after sterilization.

## Background

Digital Surgical Planning (DSP) is widely used for the presurgical design of complex cases in orthopedic, orthognathic, and facial reconstructive surgery, among others [1–5]. The DSP provides the surgeon with an opportunity to plan, calculate, and predict surgical complications, avoiding improvisations during the procedure [1, 3–5].

Medical 3D printing [6] is used pre-operatively. Anatomic models are used as part of the DSG along with anatomic guides [7]. Overall quality and accuracy [8, 9] includes dimensional fidelity.

Material Extrusion is one of the seven 3D printing methods commonly used in the medical sector. Commerical and other Term Examples include Fused Deposition Modeling (FDM) and Fused Filament Fabrication (FFF) [10].

One 3D printed using material extrusion is acrylonitrile-butadiene-styrene (ABS) [2, 3, 11–13]. ABS has good resistance, strength, and stiffness [1, 3–5, 14].

Whenever a 3D printed part is brought to a sterile field and used for intervention – for example, as a surgical guide – four properties must be considered: sterility, biocompatibility, mechanical properties, and geometric fidelity. Sterility tests would include a biological study before and after sterilization and were not performed as part of this project. The biocompatibility of the base material is extremely important in choosing a suitable material for surgical use. When using a material that will contact human tissue, ISO 10993 should be used to determine which tests may be suitable for the specific tissue contact and contact duration. A material may be rendered “sterile” but be completely un-biocompatible and therefore may harm human tissue. Studying the material’s biocompatibility after sterilization is crucial because many materials change during sterilization, including low-temperature processes such as hydrogen peroxide plasma. If a surgical guide is 3D printed, the mechanical properties must be demonstrated before use; this becomes less important if an anatomic model is used in the surgical field. Finally, it is essential to determine if the sterilization changes the geometry of the 3D part and how much change is identified.

This project focuses on the geometry of 16 example parts printed in ABS using Material Extrusion and sterilized with VHP. This project makes the assumption that the models and guides printed in ABS must be sterilized [11, 13, 15] as one of the four prerequisites for when a 3D printed part is brought to a sterile field and used for intervention. ABS devices can be sterilized by low-temperature with VHP, a method appropriate for sensitive instruments as the temperature cycles do not exceed 50 °C [11, 13, 15]. However, whether or not the sterilization process could influence the geometrical properties and affect the anatomic models and guides’ precision and accuracy is still under consideration.

The purpose of this study was to evaluate the dimensional stability of 3D printed anatomic models and guides designed using the Mimics Innovation Suite and manufactured in ABS before and after the low-temperature sterilization process. A second objective was to evaluate the accuracy of the models and guides to the original design.

## Methods

### Anatomic models and guides design

The precision and dimensional stability were analyzed in two separate processes: one for the anatomic models and one for the guides.

De-identified CT scan Digital Imaging and Communication in Medicine (DICOM) files from 8 patients with maxillary and mandibular defects were used to design the anatomic models. Eight patients with mandibular defects that needed correction using fibula segments were utilized for the guides.

**Fig. 1:**
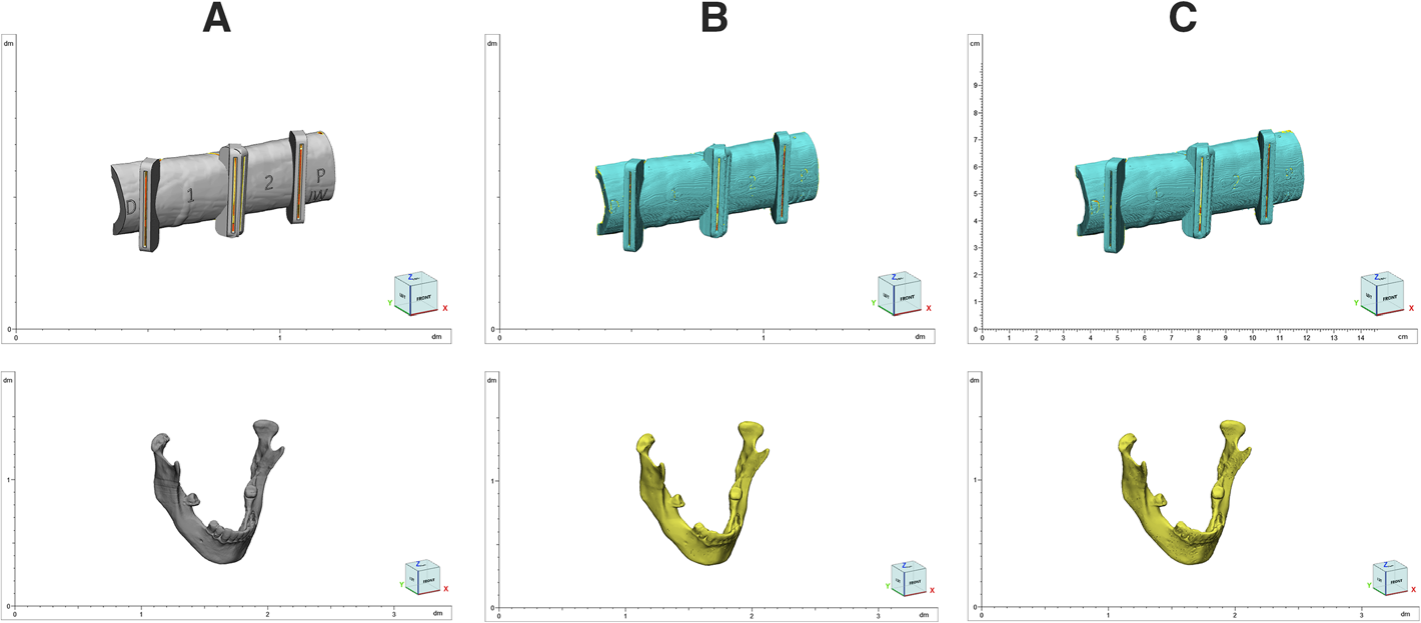
STL files. The image depicts the STL files of models (superior row) and guides (inferior row) of the original design (**A**) and the printed piece before (**B**) and after (**C**) the sterilization process

All the DICOM files from the CT scans were imported into the Mimics Innovation Suite (Materialise NV, Leuven, Belgium) for the segmentation of the images and conversion into virtual 3D models in the stereolithography (STL) format (Fig. 1A). According to the DSP in each case, the needed subdivisions of the 3D anatomic model and design of the guides were also performed.

### 3D printing and processing

A print code (G code) was generated from the STL files, and the parts were 3D printed on an Ultimaker S5 desktop material extrusion 3D printer (Ultimaker B.V., the Netherlands) in medical-grade ABS of 1.75 mm from Smart Materials 3D (Spain; ISO 10993-1 certification of biocompatibility with the human body).

The surgical guides were printed at 0.15 mm resolution and 25 mm/s speed, with extruder and build plate temperatures of 250 °C and 80 °C, respectively, and 45 degrees of minimum support overhang angle. For the anatomical models, the resolution and speed were 0.2 mm and 60 mm/s, respectively. The extruder and build plate temperatures were 250 °C and 85 °C. The minimum support overhang angle was 60 degrees.

A high-resolution scanning protocol with the CT-scanner Bright Speed Elite (General Electric; Boston, Massachusetts, USA) was used to scan the anatomic models, and the 3D optical scanner Atos Core 80 with 0.03 mm resolution (GOM, ZEISS Group, Braunschweig, Germany) for the scanning of the guides after the 3D printing (Fig. 1B).

### Sterilization process

The medical-grade ABS devices were subjected to low-temperature sterilization with VHP using a V-PRO® 1 Low-Temperature Sterilization System (STERIS Corporation, Mentor, OH), with the non-lumen cycle at 50 °C temperature.

3D scans of the models and guides were taken after the sterilization process. The scanning protocol for the models and guides was repeated after the sterilization (Fig. 1C).

### Biocompatibility evaluation

The Biological Evaluation was performed according to ISO 10993-1:2018, ISO 7405:2018, and the FDA Guidance document “Use of International Standard ISO 10993, ‘Biological Evaluation of Medical Devices Part 1: Evaluation and Testing within a Risk Management Process” [16]. It included the physicochemical material characterization (UL International GmbH/Eurofins BioPharma Product Testing Munich GmbH, Germany) and the cytotoxicity, sensitization, and material-mediated pyrogenicity tests (Nelson Laboratories, LLC. A Sotera Health Company, USA). The microbiological contaminants were also evaluated: bacterial endotoxins (according to USP < 85>) and bioburden (according to EN ISO 11737-1:2018), all performed by Nelson Laboratories, LLC. A Sotera Health Company, USA.

### Dimensional stability and statistical analysis

**Fig. 2:**
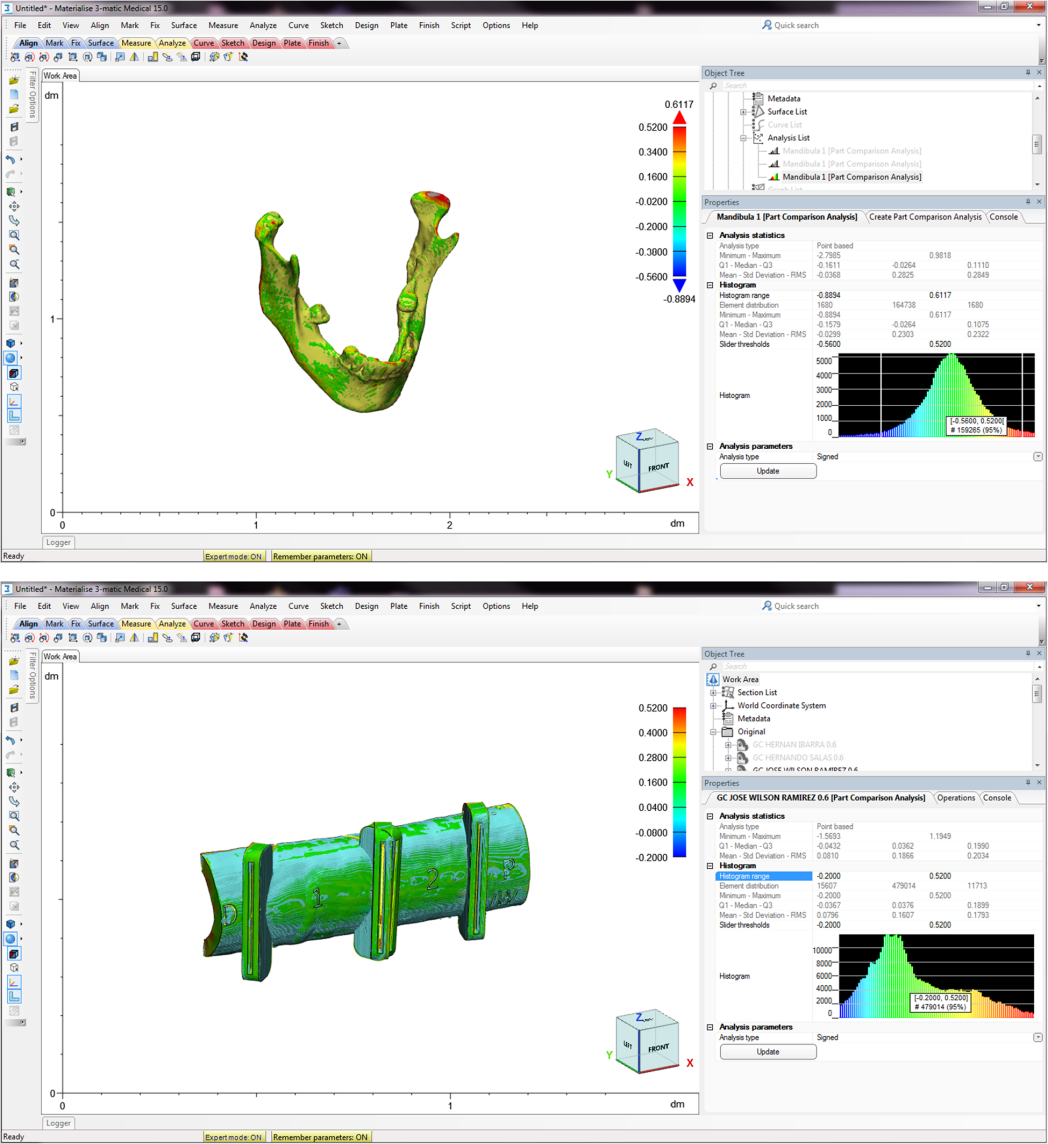
STL files superimposition and comparison. The alignment of STL files of the original design of an anatomic model and a guide with those of the printed and sterilized pieces is shown. The image displays the software tools used to analyze the alignment and deviations of the dimensions based on the histogram and the color maps

The dimensional error was calculated using the Analyze toolbox of the Mimics Innovation Suite. Three different sets of comparations were analyzed:

- “Comparison1” corresponded to the original design versus the scans made before sterilization.
- “Comparison2” corresponded to the comparison of the 3D-printed models and guides before and after sterilization.
- “Comparison3” corresponding to the original design versus the scans after sterilization (Fig. 2).

The STL files from the scans acquired before and after the sterilization process were digitally aligned, overlapped, and compared to the original design files. The dimensional error was estimated by comparing the difference between the overlapped images on a point-by-point basis; the distance amongst the points in the different coordinates in all the planes: X, Y, and Z, indicated the error. The software displayed these differences through a “color map” on the scanned model and guide.

Statistical analyses were performed with the IBM SPSS Statistics 25 software (Chicago, IL). All parameters were measured in millimeters (mm). After evaluating each data set’s distribution, the averages, standard deviations (SD), and the 95% confidence intervals (95%CI) of the “Comparison2” differences were calculated and plotted to test the dimensional stability of the 3D-printed pieces after sterilization. The same applied to both “Comparison1” and “Comparison3” to evaluate the sterilized pieces’ dimensional accuracy related to the original design. Additionally, a paired t-test was used to estimate the differences in mean distances (differences) after each process and the correlation between those values. A *p*-value of less than 5% was considered significant.

## Results

### Dimensional stability of sterilized 3D-printed models and guides

**Fig. 3:**
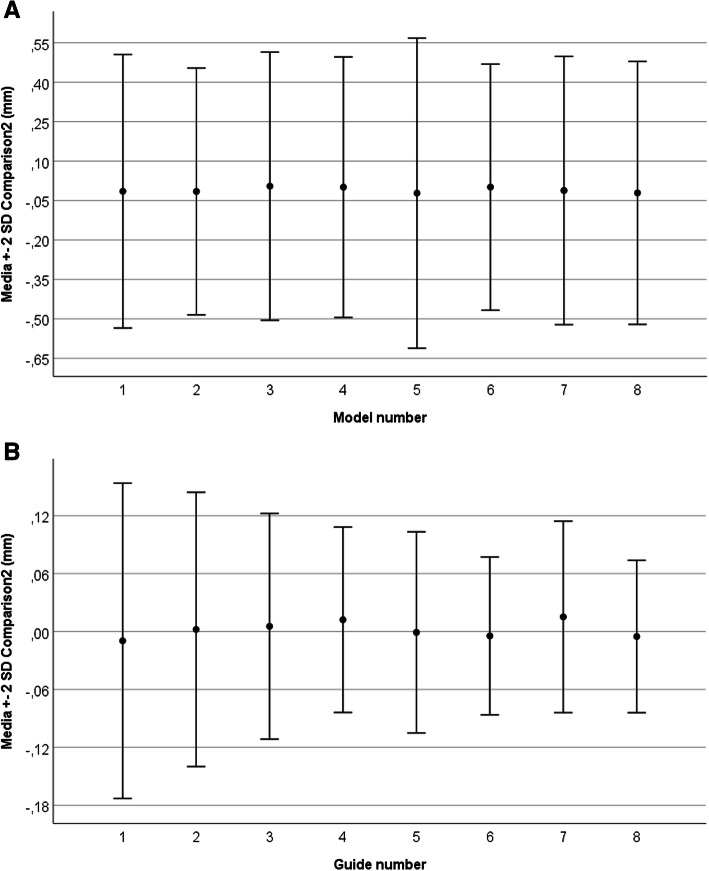
Dimensional stability before and after sterilization. The figure shows the mean differences in the alignment and comparison of STL files of the 3D-printed pieces before and after the sterilization process (“Comparison2”). The error bars indicate two standard deviations

In the “Comparison2” of the 3D-printed models before and after sterilization, the average of the estimated mean differences was − 0,011 ± 0,252 mm (95%CI − 0,011; − 0,010). In this data set, the largest mean difference between the points of the superimposed scans of pre-sterilized and post-sterilized models was − 0,022 ± 0,295 mm (95%CI − 0,025; − 0,019). Regarding the guides, the average of the estimated mean differences for the “Comparison2” was 0,003 ± 0,057 mm (95%CI 0,002; 0,003). In this series, the largest mean difference was 0,015 ± 0,050 mm (95%CI 0,015; 0,015). The mean differences between the non-sterilized and sterilized 3D-printed pieces (models and guides) are displayed in Table 1, and the median trends are in Fig. 3.

**Table. 1:** Estimated differences between the non-sterilized and sterilized 3D-printed pieces (Comparison2)

	Measured points (n)	Mean difference^a^	SD^a^	Lower 95%CI^a^	Upper 95%CI^a^
**Model Number**					
1	33615	−0,015	0,260	−0,018	− 0,012
2	42059	−0,016	0,235	−0,018	−0,013
3	33125	0,004	0,255	0,002	0,007
4	35558	0,001	0,248	−0,002	0,003
5	33908	−0,022	0,295	−0,025	−0,019
6	76740	0,001	0,234	−0,001	0,003
7	76551	−0,012	0,255	−0,014	−0,010
8	91502	−0,021	0,250	−0,023	−0,019
**Total**	**423058**	**−0,011**	**0,252**	**−0,011**	**−0,010**
**Guide Number**					
1	47785	−0,010	0,082	−0,010	− 0,009
2	116214	0,002	0,071	0,002	0,003
3	192570	0,005	0,058	0,005	0,006
4	73695	0,012	0,048	0,012	0,012
5	138000	−0,010	0,052	−0,001	−0,001
6	108202	−0,005	0,041	−0,005	−0,004
7	69266	0,015	0,050	0,015	0,015
8	23114	−0,005	0,039	−0,006	− 0,005
**Total**	**768846**	**0,003**	**0,057**	**0,002**	**0,003**

^a^Values are expressed in millimeters. *SD* Standard deviation, *CI* Confidence interval

### Dimensional accuracy of sterilized 3D-printed models and guides

In the “Comparison1” and “Comparison3” of the original design vs. the 3D-printed models before and after sterilization, the averages of the estimated mean differences were − 0,095 ± 0,536 mm (95%CI − 0,096; − 0,094) and − 0,082 ± 0,626 mm (95%CI − 0,083; − 0,081), respectively. The largest mean differences between the points of the superimposed scans of “Comparison1” and “Comparison3” were − 0,168 ± 0,469 mm (95%CI − 0,170; − 0,166) and − 0,179 ± 0,737 mm (95%CI − 0,182; − 0,176). Regarding the guides, the averages of the estimated mean differences for the “Comparison1” and “Comparison3” were 0,141 ± 0,240 mm (95%CI 0,140; 0,141) and 0,126 ± 0,205 mm (95%CI 0,126; 0,127), respectively. The largest mean differences were 0,244 ± 0,355 mm (95%CI 0,244; 0,245) for Comparison1” and 0,238 ± 0,259 mm (95%CI 0,237; 0,240) for Comparison3”. The mean differences between the original design and scans of non-sterilized and sterilized 3D-printed parts (models and guides) are displayed in Table 2.

**Table. 2:** Estimated differences between the original design and the 3D-printed pieces before (Comparison1) and after the sterilization process (Comparison3)

	Measured points (n)	Mean difference Comparison1^a^	SD^a^	Lower 95%CI^**a**^	Upper 95%CI^**a**^	Mean difference Comparison3^a^	SD^a^	Lower 95%CI^a^	Upper 95%CI^a^
**Model Number**									
1	168098	−0,019	0,467	−0,021	− 0,017	0,017	0,529	0,015	0,020
2	144685	−0,037	0,398	−0,039	−0,035	− 0,023	0,452	− 0,025	−0,020
3	242098	−0,168	0,469	−0,170	−0,166	− 0,165	0,516	− 0,167	−0,163
4	114841	−0,072	0,529	−0,075	−0,069	− 0,071	0,623	− 0,073	−0,066
5	100099	0,021	0,570	0,017	0,024	0,074	0,719	0,069	0,078
6	128057	−0,111	0,506	− 0,114	−0,108	− 0,075	0,630	− 0,078	−0,071
7	215781	−0,139	0,620	−0,141	−0,136	− 0,179	0,737	− 0,182	−0,176
8	171893	−0,134	0,643	−0,138	−0,131	− 0,097	0,723	− 0,100	−0,094
**Total**	**1285552**	**−0,095**	**0,536**	**−0,096**	**−0,094**	**− 0,082**	**0,626**	**− 0,083**	**-0,081**
**Guide Number**									
1	47785	0,115	0,189	0,114	0,117	0,119	0,198	0,117	0,121
2	116214	0,091	0,207	0,090	0,092	0,100	0,194	0,099	0,101
3	192570	0,162	0,210	0,161	0,163	0,116	0,193	0,115	0,116
4	73695	0,100	0,184	0,098	0,101	0,083	0,174	0,082	0,084
5	138000	0,244	0,355	0,244	0,245	0,238	0,259	0,237	0,240
6	108202	0,118	0,188	0,117	0,119	0,092	0,175	0,091	0,093
7	69266	0,071	0,175	0,070	0,073	0,098	0,140	0,097	0,099
8	23113	0,093	0,195	0,091	0,096	0,071	0,170	0,069	0,073
**Total**	**768845**	**0,141**	**0,240**	**0,140**	**0,141**	**0,126**	**0,205**	**0,126**	**0,127**

^a^Values are expressed in millimeters. *SD* Standard deviation, *CI* Confidence interval

**Fig. 4:**
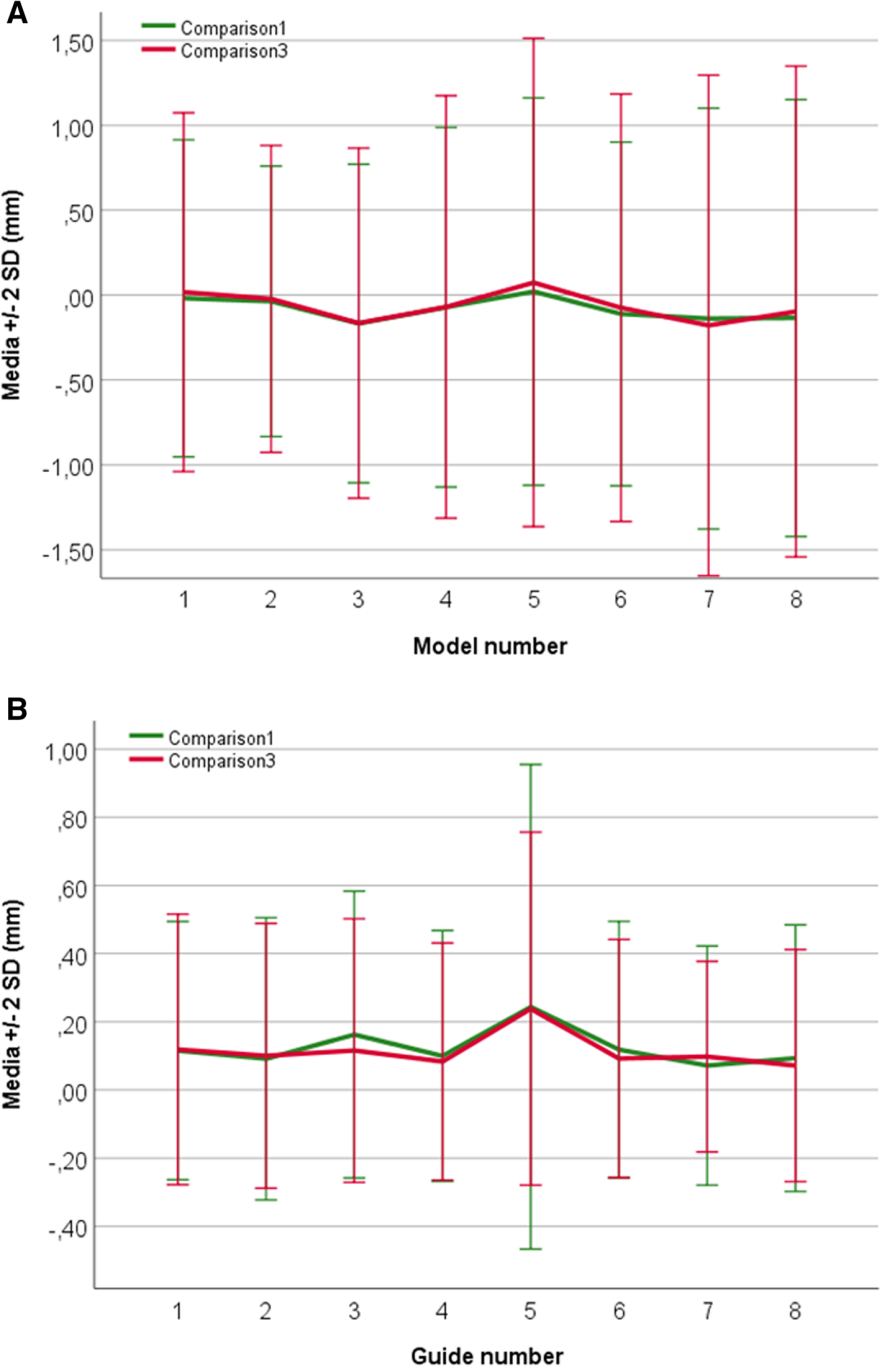
Accuracy of 3D-printed pieces after sterilization. The illustration displays the plots of the mean differences of the alignment and comparison of STL files of the 3D-printed pieces before and after sterilization with the original design (“Comparison1” and “Comparison3”). The error bars indicate two standard deviations

The mean differences between “Comparison1” and “Comparison3” for the models and guides were − 0,013 ± 0,672 mm (95%CI − 0,014; − 0,012) and 0,015 ± 0,299 mm (95%CI 0,014; 0,015). The correlations between the two sets of comparisons were of 0,399 for the models and 0,106 for the guides (both with *p* < 0,05) (Table 3 and Fig. 4).

**Table. 3:** Overall results of the dimensional differences and correlations between the three sets of comparisons

	Mean difference^a^	SD	Lower 95%CI^a^	Upper 95%CI^a^	Correlation	***P***-value
**Models**						
Comparison1 vs. Comparison2	-0,043	0,522	-0,045	-0,042	-0,004	< 0,05
Comparison2 vs. Comparison3	0,037	0,571	0,035	0,039	-0,004	
Comparison1 vs. Comparison3	-0,013	0,672	-0,014	-0,012	0,339	
**Guides**						
Comparison1 vs. Comparison2	0,138	0,247	0,138	0,139	-0,002	
Comparison2 vs. Comparison3	-0,123	0,213	-0,124	-0,123	0,013	
Comparison1 vs. Comparison3	0,015	0,299	0,014	0,015	0,106	

^a^Values are expressed in millimeters. *SD* Standard deviation, *CI* Confidence interval

### Material biocompatibility

In the biological evaluation, the anatomical models and guides showed clinically uncritical amounts of organic and inorganic leachable substances, no cytotoxic, pyrogenic, or sensitizing properties, and no acute systemic toxic potential, making them safe for the patients. The endotoxins level was below 20 EU per device, meeting the acceptance criteria, and the bioburden was less than 10^6^ colony forming units (CFU), evidencing compliance with the acceptance criteria.

## Discussion

Low-temperature sterilization is of interest for medical 3D printing, and there is a paucity of literature addressing all 4 properties: sterility, biocompatibility, mechanical properties, and geometric fidelity. In the present study, the effect of the sterilization process on the dimensional fidelity of material extrusion ABS 3D printed anatomic models and guides, and accuracy to the original design were assessed. After sterilization with VHP in both groups, models, and guides, the mean differences in dimensional stability were under ±0,5 mm and ± 0,05 mm, respectively. Likewise, the mean differences in the accuracy of the models and guides after sterilization to the original design were under ±1 mm and ± 0,25 mm, respectively. The high fidelity to the original design argues that one of the four prerequisites would be satisfied if VHP methods underwent further investigation in support of clinical use.

Anatomic models benefit surgical preparation, training, and education [6, 7]. When used in the operating room as a visual aid, 3D printed parts could come in contact with the patient [17–20].

Material extrusion printed models and guides are prone to contraction and distortion during the thermoplastic cooling process, leading to geometric inaccuracies [8]. Several studies have assessed the dimensional accuracy of material extrusion pieces, biomedical and non-biomedical, manufactured in ABS after the 3D printing process. Popescu et al. [13] evaluated several dimensions of a non-biomedical ABS test part, measuring and comparing it to the nominal values in different sections. They found divergence values of +/− 0.27 mm with mostly positive deviations in comparison with the nominal part. On the other hand, E-Katatny et al. [20] and Hsu et al. [21], using anatomic models of a mandible and a canine fibula, respectively, found surface deviations of 0,159 mm and 0,121 mm to the original design. In the present study, the mean differences between the printed pieces and original design were within the 95%CI of − 0,096 to − 0,094 mm for models and 0,140 to 0,141 mm for guides. The current data closely resembles these prior studies.

While material extrusion produces devices with some degree of sterility given the high temperatures [22], printing itself is insufficient to meet intraoperative criteria. Moreover, non-sterile handling contaminates the devices, would be considered unsafe and leads to an intraoperative infection [11].

The sterilization methods adequate for different 3D printing materials have been tested in terms of infection rate and dimensional stability, or, on the contrary, geometrical deformation [11, 23–26]. Low-temperature VHP has been used for sterilization of printouts produced by FDP in ABS. They show a low infection rate with the preservation of the geometrical dimensions [11, 13, 21]. Popescu et al. [13] demonstrated that low-temperature sterilization with VHP did not influence the dimensions of ABS specimens and the geometrical features remained stable [13]. However, it has been indicated that for more multifaceted structures, mainly containing large surfaces of low depth, sterilization with VHP could significantly impact the accuracy [27].

Two-dimensional factors are essential when considering the use of a model or guide after the sterilization process. First, the stability, in which the mean surface deviations of the pieces do not considerably alter the proportions, making them suitable for their use in the operating room and patients. Second, the devices’ accuracy with a high level of conformity concerning the original design being truthful to the patient’s anatomy [21, 26, 27].

The structural variations of VHP sterilized pieces produced in ABS have been previously addressed in various publications. As mentioned above, Popescu et al. [8] assessed a non-biomedical ABS part for dimensional accuracy following the printing and sterilization processes. After the latter, the dimensional changes were +/− 0,20 mm, leading the authors to conclude that the sterilized part’s dimensions were closer to the nominal design than the pre-sterilized one. Likewise, Kuczko et al. [27] evaluated the effect of VHP sterilization over non-biomedical ABS 3D-printed pieces finding an average dimensional error of 0,036 mm [27]. Hsu et al. [21] also tested the effect of low-temperature sterilization on the canine fibula model, getting a mean deviation of 0,043 mm [21].

The biological evaluations showed that the anatomical models and surgical guides were biocompatible, that the contaminants were effectively removed from the surface, and the microbiological load was low and according to the standards.

This manuscript does not study or prove the biocompatibility of ABS after VHP.

The study has some limitations and should not be interpreted out of context for clinical use.

The most critical limitation is that while our study address with statistical rigor geometric fidelity, the other factors have not undergone rigorous study, although there is preliminary data supporting biocompatibility. The current data and report are not intended to supplant regulatory approval for clinical use.

Overall, it is not expected that the lower temperatures will deform a part that is 3D printed using material extrusion; this is confirmed by the data presented. There would be several additional studies that would be required to support clinical use. For example, mechanical strength was not evaluated, and sterility validation was outside the scope of the present study.

The second limitation resides in that testing only uses one material and one 3D printer. There may exist other conditions under which these results are not reproducible. Third, the dimensional analysis was performed by one engineer, which in theory could have introduced some bias on the measurements. However, the software provides programmed alternatives that assist in the device’s alignments, eliminating manual errors and biases.

## Conclusion

The dimensional stability of 3D printed anatomic models and guides designed using the Mimics Innovation Suite and 3D printed in ABS using material extrusion was not affected after low-temperature sterilization with Vaporized Hydrogen Peroxide. Further work will require additional data regarding sterility, biocompatibility, mechanical properties to support potential clinical use.

## Data Availability

The datasets generated and analyzed during the current study are not publicly available due to corporate policies but are available from the corresponding author on reasonable request.

## Abbreviations

DSP: Digital Surgical Planning
3D: Three-dimensional
FDM: Fused deposition modeling
ABS: Acrylonitrile-butadiene-styrene
VHP: Vaporized hydrogen peroxide
CT: Computer tomography
STL: Stereolithography
SD: Standard deviations
